# Identifying a novel powdery mildew resistance gene in a barley landrace from Morocco

**DOI:** 10.1007/s13353-019-00505-y

**Published:** 2019-07-17

**Authors:** Urszula Piechota, Paweł C. Czembor, Piotr Słowacki, Jerzy H. Czembor

**Affiliations:** Plant Breeding & Acclimatization Institute – National Research Institute, Radzików, 05-870, Błonie, Poland

**Keywords:** Barley (*Hordeum vulgare* L.), DArTseq markers, Powdery mildew (*Blumeria graminis* f. sp. *hordei*), Resistance gene, Linkage mapping

## Abstract

Powdery mildew is a barley foliar disease that causes great loss in yield. Because of the limited number of effective resistance genes, efforts to identify new sources of resistance are frequently focused on genetically diversified landraces. The goal of this study was to characterise the powdery mildew resistance gene in barley line 2553-3 selected from the Moroccan landrace. Phytopathological testing against a set of differential pathogen isolates revealed different pattern responses of this gene from those of other known resistance genes. F_2_ and F_2:3_ (2553-3 × Manchuria) mapping populations were employed to investigate resistance inheritance. Two approaches were applied for the linkage analysis: in the first approach, 22 resistant and 21 susceptible homozygous F_2_ plants genotyped by the DArTseq platform (Diversity Arrays Technology, Pty. Ltd.) were used; in the second, 94 F_2_ plants were genotyped by converted DArTseq markers and SSRs. Both analyses delineated a new resistance gene on the short arm of chromosome 2H. The authors propose *MlMor* as a gene symbol for newly characterized powdery mildew resistance genes in barley line 255-3-3. The results presented herein provide a good foundation for the development of closer linkage markers and MAS breeding.

## Introduction

Barley (*Hordeum vulgare* L.) is the second most important cereal according to harvest area in Europe (FAOSTAT 2016. http://www.fao.org/faostat) and is generally grown as fodder and for brewing purposes. Although barley has low environmental requirements and can be farmed under harsh conditions (Nevo 1992; Stanca et al. 1992; Newton et al. 2010; Honsdorf et al. 2014), fungal infections are a cause of concern. *Blumeria graminis* (D.C.) Golovin ex Speer f. sp. *hordei* Em. Marchal (*Bgh*) is an obligate Ascomycota pathogen that causes powdery mildew, one of the most widespread foliar diseases. Infection by this fungus leads to yield loss and decreased feed and malting quality. The two common approaches of controlling epidemics involve the use of fungicides and the cultivation of resistant crops. Chemical protection can provide positive selection for pathogen fungicide-resistant strains (Lucas et al. 2015), whereas the cultivation of resistant crop varieties is among the most economically effective and environmentally friendly methods of controlling disease.

Resistance genes for powdery mildew are widely distributed on the barley genome. Known resistance genes mapped on the barley genome include *Mla* - with almost 30 alleles, as well as five other genes—*Mlat*, *MlGa*, *Mlk*, *Mlnn*, and *Mlra*—on the chromosome 1H; *MlLa* on 2H; *mlo*, *Mlg*, and *MlBo* on 4H; *Mlj* on 5H; *Mlh* on 6H; and *mlt* and *Mlf* on 7H (Jørgensen and Wolfe 1994; Schönfeld et al. 1996). Except for *mlo*, all of these genes are race-specific, major resistance genes and subject to the “boom and bust effect,” with newly emerging pathotypes eventually overcoming resistance within a few years. The most effective and durable resistance depends on *mlo*, a recessive allele originating from Ethiopian landraces (Büschges et al. 1997) that has been widely introduced into European cultivars. However, the introduction of *mlo* can generate some negative outcomes: Mlo-resistant varieties are more susceptible to necrotrophic and hemibiotrophic pathogens (Brown and Rant 2013), and *mlo* has an impact on yield by decreasing the thousand-grain weight and yield from a given plot due to pleiotropic effects (Kjær et al. 1990).

Because of the limited number of known resistance genes utilised to control barley powdery mildew, new potential resources need to be identified. Barley landraces originate from regions with traditional and primitive farming systems, which lack explicit crop progress (Camacho Villa et al. 2005). These materials are promising sources of genetic diversity. Landraces comprise heterogenic dynamic populations subject to natural selection. Long-term co-evolution of a host with the fungus that causes powdery mildew provides low pathogen pressure and increases durability and polymorphism at resistance gene loci. Previous screening studies on powdery mildew resistance in barley landraces have revealed novel resistant lines (Comadran et al. 2009). For example, Czembor (2000, 2002) investigated 131 barley lines originating from Moroccan landraces. During phytopathological testing, previously unknown powdery mildew resistance genes were found in 66 lines, which showed unique resistance reaction spectra that were distinguishable from those of other known genes. Among these lines, only 255-3-3 was resistant to all 23 *Bgh* isolates studied and it exhibited no visible infection symptoms to most (74%) of them (Czembor 2002). The aim of the present study was to identify resistance genes in barley line 255-3-3.

## Materials and methods

### Plant materials

Spring barley (*H. vulgare*) line 255-3-3 (National Centre for Plant Genetic Resources Plant Breeding and Acclimatization Institute, Poland, https://bankgenow.edu.pl, ID number 17I00361) was used for identifying powdery mildew resistance genes. This line was selected from Moroccan landrace 255 (ICARDA No. ICB 31956) and showed broad-spectrum resistance to powdery mildew in a previous study (Czembor 2002).

The mapping population was derived from a cross of 255-3-3 as the female parent with susceptible cv. Manchuria as the male parent.

An F_2_ population derived from a cross of 255-3-3 with P23 (*MlLa*) was used for allelic testing.

A set of 30 differential varieties (DV) carrying various known powdery mildew resistance genes was used for the phytopathological tests. This set contained cv. Pallas, 21 Pallas near-isogenic lines (Kølster et al. 1986) and 8 selected cultivars (Table 1). This DV set represented most of the major resistance genes used in European cultivars.

**Table 1:** Infection types of differential varieties and the 255-3-3 line after inoculation by with a set of *Blumeria graminis* f. sp. *hordei* isolates according to the Mains and Dietz scale (1930) extended by 0(4) point indicates Mlo resistance

Variety	Resistance genes	*Blumeria graminis* f. sp. *hordei* isolate accession numbers																								
		1	3	13	18	19	20	24R	25	26	27	34	38	48	50	51	111	123	129	131	133	1-26	2-9	3-52	3-55	3-60
255-3-3	u	1	2	2	2	2	1	1	2	2	0	2	2	2	2	1	1	2	2	2	2	1	1	1	1	1
Manchuria	–	4	4	4	4	4	4	4	4	4	4	4	4	4	4	4	4	4	4	4	4	4	4	4	4	4
Pallas	*Mla8*	4	4	4	4	4	4	4	4	4	4	4	4	4	4	4	4	4	4	4	4	4	4	4	4	4
P01	*Mla1*	0	4	0	0	0	0	0	0	0	0	0	0	0	0	4	0	4	0	4	4	4	4	4	4	4
P02	*Mla3*	0	0	1	0	0	0	4	0	0	0	1	0	0	0	1	0	0	0	4	4	0	4	4	4	2
P03	*Mla6, Mla14*	0	0	0	4	4	0	4	0	0	0	0	0	4	4	4	4	4	4	4	4	4	4	4	4	2
P04B	*Mla7, Mlk*	4	4	4	4	4	4	4	4	0	2	4	0	0	4	4	4	4	4	4	4	4	4	4	4	4
P06	*Mla7, MlLG2*	4	4	4	4	4	1	4	4	0	0	4	0	0	4	4	2	4	4	2	4	2	4	4	4	4
P08B	*Mla9*	4	4	4	0	0	0	0	4	4	0	0	0	0	4	0	4	4	4	0	0	4	0	0	0	4
P09	*Mla10, MlDu2*	4	4	4	0	0	4	0	4	4	4	0	1	0	4	4	4	4	4	4	0	4	4	0	1	1
P10	*Mla12*	0	4	0	4	2	4	0	0	0	0	4	0	0	0	4	4	4	4	4	4	4	4	4	0	2
P11	*Mla13, MlRu3*	4	4	0	4	4	0	0	0	4	0	4	0	0	0	4	4	4	4	0	4	1	0	4	4	0
P12	*Mla22*	4	0	0	4	4	0	4	0	0	0	4	4	4	0	4	0	0	4	4	4	1	0	4	4	4
P13	*Mla23*	4	1	2	2	2	2	1	2	2	2	2	2	4	2	4	1	1	1	1	1	4	1	1	1	2
P14	*Mlra*	4	4	4	4	4	4	4	4	4	4	4	4	4	4	4	4	4	4	4	4	4	4	4	4	4
P15	*Ml(Ru2)*	2	4	2	4	4	4	4	4	2	2	4	2	4	4	4	4	4	4	4	2	4	4	4	4	4
P17	*Mlk*	4	4	4	2	2	2	1	4	4	0	1	0	0	4	4	4	4	4	4	2	4	4	4	2	2
P18	*Mlnn*	4	4	4	4	4	4	2	4	2	2	4	4	4	4	4	4	4	2	4	2	4	2	4	4	4
P19	*Mlp*	2	2	0	2	2	2	2	2	2	2	2	2	2	2	2	2	2	2	2	2	2	2	2	2	1
P20	*Mlat*	2	2	4	2	2	2	2	4	2	2	2	2	2	2	4	2	2	2	2	4	4	2	2	2	2
P21	*Mlg, Ml(CP)*	4	4	0	0	0	0	4	4	4	4	4	4	0	0	4	4	4	4	2	4	4	0	4	4	4
P22	*mlo5*	0(4)	0(4)	0(4)	0(4)	0(4)	0(4)	0(4)	0(4)	0(4)	0(4)	0(4)	0(4)	0(4)	0(4)	0(4)	0(4)	0(4)	0(4)	0(4)	0(4)	0(4)	0(4)	0(4)	0(4)	0(4)
P23	*Ml(La)*	4	4	4	4	4	4	4	4	4	4	4	4	4	4	4	4	4	2	4	2	2	2	4	4	2
P24	*Mlh*	4	4	4	4	4	4	4	4	4	4	4	0	4	4	4	4	4	4	4	4	4	4	4	4	4
Benedicte	*Mla9, Ml(IM9)*	0	4	0	0	0	0	0	0	0	0	4	0	0	0	4	4	4	4	4	4	NA*	0	4	0	1
Lenka	*Mla13, Ml(Ab)*	0	4	0	2	4	0	0	0	2	0	4	0	0	0	4	4	4	4	0	4	NA	0	4	4	0
Gunnar	*Mla3, Ml(Tu2)*	0	0	0	0	0	0	0	0	0	0	0	0	0	0	2	0	0	0	0	0	NA	1	1	1	0
Steffi	*Ml(St1), Ml(St2)*	0	3	0	0	0	0	0	3	0	0	3	0	0	0	2	4	4	4	4	2	NA	0	4	4	1
Triumph	*Mla7, Ml(Ab)*	4	0	0	4	4	0	4	4	4	4	4	4	4	4	4	4	4	4	4	4	NA	4	4	4	2
Borwina	*Ml(Bw)*	2	2	2	4	4	4	4	4	2	1	4	2	3	4	4	4	4	4	4	1	NA	4	2	4	1
Iron	*Ml(1-B-53)*	0	0	0	0	0	0	0	0	0	0	0	0	0	0	0	0	4	0	0	0	NA	0	0	0	0
Souleyka	*Ml(Lv)*	NA	NA	NA	NA	NA	NA	NA	NA	NA	NA	NA	NA	NA	NA	NA	NA	NA	NA	NA	NA	NA	4	1	4	0

*)Not available data

Plants were grown in a control environment under a 19 °C/15 °C (16-h day/8-h night) regime. For *Bgh* propagation and the phytopathological tests, the plants were grown in transparent boxes to prevent mildew contamination.

### Phytopathological tests

A set of 25 *Bgh* isolates collected in Poland in 2010, 2015 and 2017 were obtained from the collection of Plant Breeding and Acclimatization Institute, Poland. *Bgh* isolates were selected to achieve differences in virulence spectra and to specify the presence of resistance genes among the DV (Table 1).

Fungal inoculum was freshly propagated on susceptible cv. Manchuria. Ten-day-old seedlings with fully expanded first leaves were inoculated by shaking conidia from diseased plants. On the 8th day after inoculation, infection types (ITs) were scored on a 0–4-point scale (Mains and Dietz 1930), where 0, 1, and 2 indicate resistance, and 3 and 4 indicate susceptibility; extended by 0(4) level indicates Mlo resistance.

To determine the resistance of 255-3-3, the ITs of this line after inoculation with the set of *Bgh* isolates were compared with those obtained for the DV set. Tests were conducted with ca. 15 seedlings per line in two repetitions.

To determine the inheritance of resistance of 255-3-3, 190 F_2_ 255-3-3 **×** Manchuria plants and 128 F_2:3_ families (25 individuals per family) were inoculated with the isolate *Bgh*27 avirulent to 255-3-3.

For allelic testing, 315 F_2_ 255-3-3 × P23 plants were inoculated with isolate *Bgh*1-26 avirulent to both parental lines.

The numbers of resistant and susceptible plants were compared to those expected based on the theoretical Mendelian segregation ratio by the chi-square (χ^2^) test (*p* = 0.05).

### Molecular analysis

Genomic DNA from 94 plants from F_2_ 255-3-3 **×** Manchuria and the parental lines was used for the molecular analysis. DNA was extracted from a single plant leaf using the CTAB method (Murray and Thompson 1980). Samples from 43 homozygous F_2_ plants (22 homozygous resistant and 21 homozygous susceptible) and from the parental lines were genotyped using the DArTseq platform (Diversity Arrays Technology, Pty. Ltd.) (Von Cruz et al. 2013), and the DArTseq data were used for linkage analysis. For that purpose, DArTseq markers that were low quality, homozygous and had >20% missing calls were removed. The remaining markers were assessed for compatibility with the resistance/susceptibility trait. Markers with > 80% goodness of fit were evaluated by Fisher’s exact test on 2 **×** 2 count tables using R (www.r-project.com). The null hypothesis was a random distribution of DArTseq marker variants within resistant and susceptible plants. Significant markers according to F-test results were assigned to a genetic location by BLASTN (Altschul et al. 1997) against barley genome Hv_IBSC_PGSB_v2 on the EnsemblPlants database release 37 (www.plants.ensembl.org, accessed 28.11.2017) (Aken et al. 2017). Alignments with a BLASTN *E* value < 1.0E^−10^ and with a minimum difference > 1.0E^−5^ between the first and second hits were selected for genotyping of 94 individuals from the F_2_ population using DArTseq markers that were converted to allele-specific dominant PCR (AS-DArT) markers. Extended DArTseq sequences from the BLASTN results were used to design allele-specific primers in BatchPrimer 3.0 (You et al. 2008) or manually without additional mismatch at the -3′ position. The nucleotide at the 3′-end of the forward primer or the 5′-end of the reverse primer was in the SNP locus. For silicoDArT markers, allele-specific primers were designed for all six potential SNPs located in the *Pst*I restriction enzyme recognition sequence (5′-C|TGCAG-3′). The *Pst*I enzyme was used for the DArTseq pipeline, and the sequencing reads were consistently generated from the *Pst*I site. Other primers were designed by Primer BLAST NCBI (Ye et al. 2012). PCR products were amplified using modified DNA polymerase SNPase (GeneON GmbH, Germany) in accordance with the manufacturer’s protocol. In addition, a set of 57 SSR markers was selected from the GrainGenes database (https://wheat.pw.usda.gov, access 11.2017) according to known localization on a chromosome of interest. SSRs were employed to genotype the F_2_ plants and parental lines. The PCR amplified fragments were separated by 1.5% agarose gel electrophoresis and visualised with ethidium bromide; fluorescently labelled fragments were detected on 4.5% polyacrylamide gels using an ABI377XL genetic analyser (Applied Biosystems, USA).

### Linkage analysis and genetic mapping

Genetic linkage maps were constructed using the JoinMap 4.0 software (Stam 1993) under the standard calculation settings: linkages with a recombination frequency smaller than 0.45 and an LOD score higher than 1; goodness-of-fit jump threshold for removing loci of 3 and performing a ripple after adding 3 loci and the Kosambi mapping function (Kosambi 1944). The phenotypic scores for the F_2_ 255-3-3 × Manchuria population were converted to binary data according to the IT scores; specifically, 0, 1 and 2 (resistant) were recoded as 1, and 3 and 4 (susceptible) were recoded as 0. The converted scores were included in the analysis as resistance gene *RBgh255*. AS-DArT, SSR markers, and the *RBgh255* gene were used for genetic map construction, and another map was generated for silicoDArT markers with physical positions on 2H. The map positions of the DArTseq markers were compared with a barley consensus 2H map from Barleymap database POPSEQ data (http://floresta.eead.csic.es/barleymap) (Cantalapiedra et al. 2015; Mascher et al. 2013) in MapChart software (http://www.joinmap.nl) (Voorrips 2002). Kruskal-Wallis analysis of associations between the DArTseq markers and resistance was carried out in MapQTL 6 software under standard conditions (van Ooijen 2009).

## Results

### Phytopathological tests

To determine the possible resistance genes present in the 255-3-3 line, we assessed resistance against a diverse collection of 25 *Bgh* isolates and compared the results with the ITs of the DV set carrying various resistance genes and with susceptible cultivar Manchuria as a control (Table 1). The 255-3-3 line exhibited distinctive disease response patterns, with IT scores of 0, 1, and 2 according to the Mains’ and Dietz’s 5-level scale (1930). The line was resistant to all *Bgh* isolates.

Evaluation of the *Bgh*-inoculated F_2_ 255-3-3 **×** Manchuria population revealed both susceptible and resistant individuals. Furthermore, phytopathological tests of F_2:3_ families showed segregating heterozygous and non-segregating homozygous F_2_ plants. The results of chi-squared tests confirmed the expectations of 3:1 for the F_2_ population and 1:2:1 for F_2:3_ (*p* = 0.05) (Table 2).

**Table 2:** Segregation ratio and chi-square test results for the analysed populations after inoculation with *Blumeria graminis* f. sp. *hordei*

Population		*Bgh* isolate	Number of plants/families			Predicted ratio	*χ* ^2^	*p* value (*p* = 0.05)
			Res	Seg	Sus			
255-3-3 × Manchuria	F_2_	*Bgh*27	145	–	45	3:1	0.1754	0.6753
	F_2:3_	*Bgh*27	40	58	30	1:2:1	2.6875	0.2609
255-3-3 × P23 (*MlLa*)	F_2_	*Bgh*1–26	237	–	78	15:1	184.2305	0.0000

### Genotyping and genetic mapping

DArTseq analysis of 43 homozygous F_2_ 255-3-3 **×** Manchuria plants identified 3544 codominant DArTSNP markers and 8711 dominant silicoDArT markers. Of these DArTseq markers, 33 were selected for conversion to AS-DArT markers and used to genotype 94 F_2_ individuals. Based on BLASTN alignment to the barley reference genome, each is located on the 2H chromosome.

**Fig. 1:**
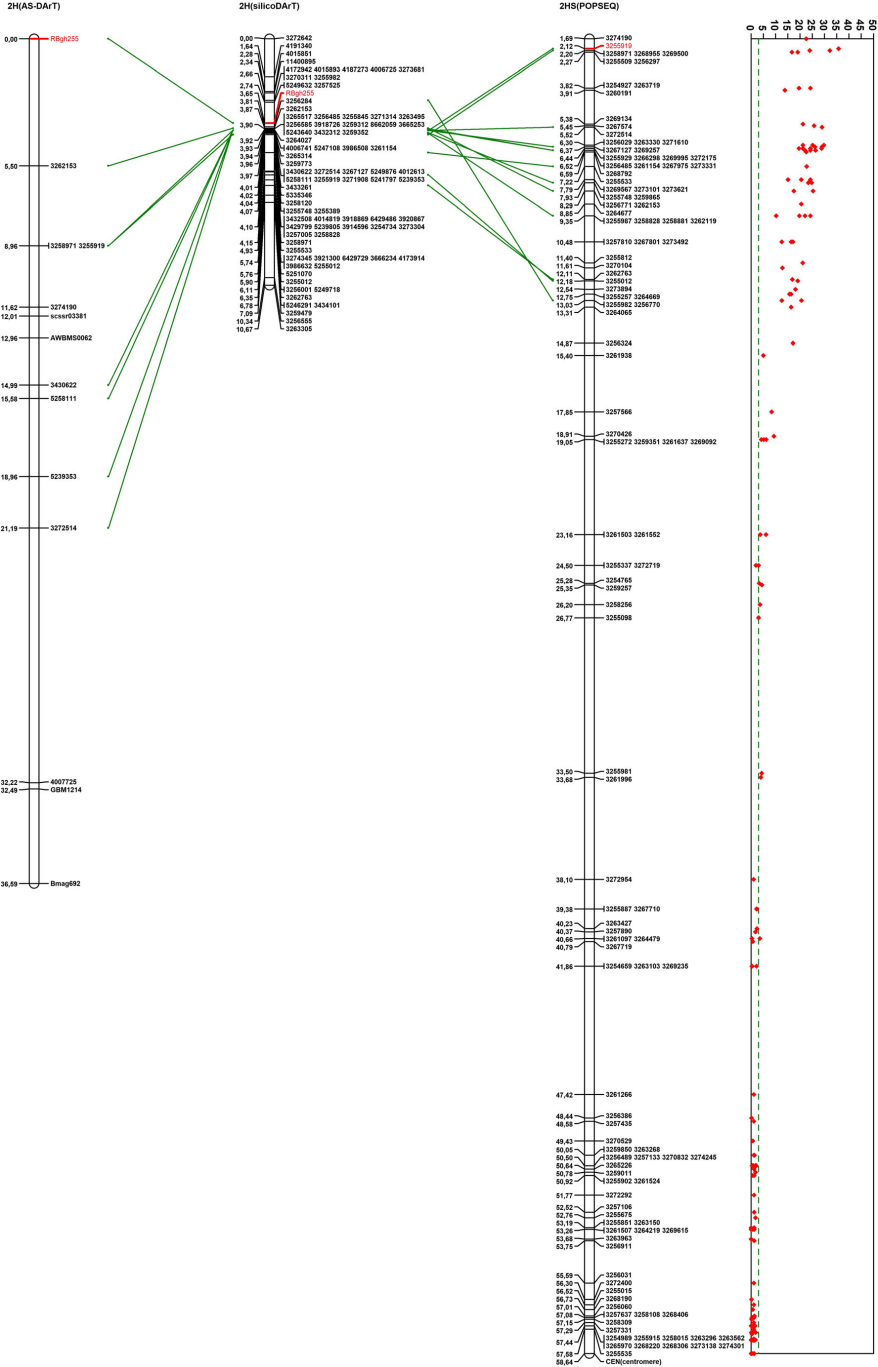
Comparative analysis of partial barley 2H maps. Comparative map analysis between the linkage map constructed for 94 F_2_ 255-3-3 × Manchuria plants (2H(AS-DArT)), the linkage map constructed for the silicoDArT data for 43 homozygous F_2_ plants (2H(silicoDArT)) and the barley consensus genetic map (2HS(POPSEQ)); Kruskal-Wallis K-statistic plot of the associations analysis between the DArTseq markers and resistance, *α* = 0.05

Linkage analysis for AS-DArT (Table 3), SSR and *RBgh255* assembled 14 markers into a single 36.59-cM group containing nine AS-DArTs, four SSRs, and *RBgh255* (Fig. 1). Based on the known position of these markers, *RBgh255* was mapped to the distal end of chromosome 2H, 5.50 cM distal to the nearest marker 3262153.

**Table 3:** DArTseq markers assembled into one linkage group with *RBgh255*

AlleleID.		Trimmed sequence	*F* test^1^*p* value	BLASTN results^2^	BLASTN *E* value	AS-Dart primer sequences
5258111	5258111|F|0-31:A>C-31:A>C	TGCAGCCACCTTGCCGGATTCACGAGCGCGGACTCCTCCTCGCCG	6.831E−09	chr2H:3653981–3654025	3E−14	F:GGATTCACGAGCGCGGA
						R:CAACGTCGGCTTCAAGCTT
5335346	5335346|F|0-37:G>A-37:G>A	TGCAGGGCTGCGTAACCTTACCAGCCTCAAGAAATTAGGAATCTGGCACTGCCCAGCAGTCTCGTCGTT	9.422E−09	chr2H:1371951–1371994	2.3E−13	F:ACCTTACCAGCCTCAAGAAATTAG
						R:TTGCAGGAAATAGCAAAGTGG
4012613	4012613|F|0-11:T>G-11:T>G	TGCAGGTACGGTCGGCTGTTCAGAACCCACATCCTGGGCTGCCCCACGGTGGTGTGCATGGACCCGAGA	4.435E−09	chr2H:4610319–4610383	2.4E−16	F:GCTGCTGCAGGTACGGG
						R:GCATGAAGGCGTCGATCT
3272514	3272514|F|0-58:C>T-58:C>T	TGCAGATATAGCAACCTCCATGGCCAGGAGTGATATGCTGGTAATGCTGCTGGTCGGGCTGTCTAGAGA	1.664E−08	chr2H:6576705–6576773	1.1E −30	F:TAATGCTGCTGGTCGGGT
						R:CAAACGTGTTACTTGTGCC
3430622	3430622|F|0-31:A>G-31:A>G	TGCAGATGTGACGACCTCGGACGTGCACGGTATGACCACTGGAACCG	5.317E−08	chr2H:8134169–8134215	8.6E −18	F:AATTGGCAAGTGCGTGCAT
						R:GCCGGTTCCAGTGGTCAT
3262153	3262153|F|0-40:G>A-40:G>A	TGCAGGCTGGATTACCGCGGCCTGCCGAGGCCCAGGCAGCGTGAGGGGCAGTTCTTGCTGGCTGAATTG	1.951E−07	chr2H:12600237–12600305	1.1E−30	F:GCTGATCACGCGACAAATG
						R:AGCAAGAACTGCCCCTCAC
5239353	5239353|F|0-37:A>C-37:A>C	TGCAGGTGGCAACGATCGACGAGAATGGCGGCCAAGGAAAACGCAAAGGGATGGTCCGTGGCCTCCGTA	6.293E−10	NA*	NA	F:AGCCATGGTATTCGACAAGG
						R:GACCATCCCTTTGCGTTTT
3274190		TGCAGTATCTCTCTCTCTCTCTCTCTCTTTTTCTGAGCATACAGTGGGATCGTTAGTCCGCTGCAATGT	7.112E−08	chr2H:4696474–4696527	1E−21	F:CATCTTTGCTTCCTGATGCC
						R:CGAGCTCCAAAACGCCTAAC
4007725		TGCAGATCCGCCGCCGTCTGACTCGTGCTGCGCAGGAGAGGCCCGACCACGGCGGCATCGGCAAGGCCT	4.40E−06	chr2H:1380942–1381004	1.1E−24	F:GCTTTGCTTCTTCGGGC
						R:CGGTCGTACCTTCCTCCGTA

^1^Fisher’s exact test; ^2^BLASTN against barley genome Hv_IBSC_PGSB_v2 on the EnsemblPlants database release 37 (www.plants.ensembl.org, accessed 28.11.2017); *not available data

The linkage map for the silicoDArT markers from 2H and *RBgh255* assembled 82 markers into one linkage 10.67-cM group (Fig. 1). *RBgh255* was mapped to 3.65 cM, 0.91 cM proximal to 5249632 and 3257525 and 0.16 cM distal to 3256284.

The Kruskal-Wallis analysis demonstrated that only DArTseq markers mapping to the distal end of chromosome 2HL were significantly associated with RBgh255 (Fig. 1); the analysis did not reveal other significant loci in the genome (data not shown). The highest *K* value (*α* = 0.05) was for 3255919. The physical position of these sequences is chr2H:1839535:1839604:1 according to the barley reference genome (Table 4). These markers were previously mapped to 2.12 cM on the 2H consensus barley map (Fig. 1). With reference to the highest *K* value marker 3255919, RBgh255 mapped 0.32 cM distal on the silicoDArT map and 8.36 cM distal on the AS-DArT map.

**Table 4:** DArTseq markers with significant K-value statistics for analysis of associations between the DArTseq markers and resistance of the barley 255-3-3 line

Locus	Group	Position	*K* value^1^	Df^2^	Sequence	Physical position^3^	*E* value^3^
3255919	2H	2.120	35.75	2	TGCAGCCTAGGGCGCTATGCACATGAACCAGGGACGTCACCCATGTAGTTGTACTATCACTTAATCAGC	chr2H:1839535:1839604:1	1.1E−30
3258971	2H	2.200	32.21	2	TGCAGGTTGAAGGAACCTGAGCCGTGCGTGAACACGAGCTGACTGGGGATGCTAACCAAGAGGCATCCG	chr2H:1320025:1320093:1	1.1E−30
3272514	2H	5.520	28.97	2	TGCAGATATAGCAACCTCCATGGCCAGGAGTGATATGCTGGTAATGCTGCTGGTCGGGCTGTCTAGAGA	chr2H:6576773:6576705:-1	1.1E−30
3267127	2H	6.370	28.97	2	TGCAGCTTGAGGTGTTAGGATTACACCACATGTGAACCAAATTTTGGCAGGTCTGTTGGTACAGCCTCA	chr2H:6807214:6807282:1	1.1E−30
3256485	2H	6.520	24.21	2	TGCAGCTCCATGCCCCCTCTTCCTCCGCCAACATCATGTCTGCCCTCATAAACCGTGACATACCCGTCC	chr2H:11687193:11687261:1	1.1E−30
						chr2H:11647629:11647561:-1	1.1E−30
3261154	2H	6.520	26.36	2	TGCAGCTTGACCTCCTCGTTGAGGAGCTGGAGCAGCTTGGGGAGCAGGGAGCCCATGGCGCCCGTTGCA	chr2H:9291206:9291138:-1	2.8E−28
3255533	2H	7.220	22.70	2	TGCAGGTTTTGTTGATGCATGATAGCTGGGTGGACCAACCAACAAAGCCTGCTATGTCGTGGTCCAGGA	chr2H:11129792:11129724:-1	1.1E-30
						chr2H:11004398:11004466:1	1.1E-30
3269567	2H	7.790	24.21	2	TGCAGCCACTAACCAAGCACTAGGCCGGCATGGCAATGGAACTGGAGAGCCTTGCCATGACTCTCCTTC	chr2H:12272164:12272232:-1	1.1E−30
						chr2H:657193730–657193798:-1	1.1E−30
3255748	2H	7.930	23.34	2	TGCAGCTGGCTCTCAACTATGTCTTCTATTGGCCAATCAAGAACATGTGTGATAGTTTCTCCGAATGGT	chr2H:13286253–13286321:1	1.1E−30
3259865	2H	7.930	24.72	2	TGCAGTCCCCGCCGACGCAGCAGCAGCAGGACCTGCTGGGCGGGCTGGACACTGAGCTGAGCGACATGC	chr2H:14052180–14052248:-1	2.8E−28
3262153	2H	8.290	25.28	2	TGCAGGCTGGATTACCGCGGCCTGCCGAGGCCCAGGCAGCGTGAGGGGCAGTTCTTGCTGGCTGAATTG	chr2H:12600237–12600305:1	1.1E−30
3258828	2H	9.350	24.21	2	TGCAGCACCCTCAGCTGCTGGTTGCGAGCCATGGTTCCCTGCTATTTTCCTGCCCGTTTGGTTTTGTTG	chr2H:14782674–14782742:1	2.8E−28
						chr2H:14859501–14859569:1	2.8E−28
3262763	2H	12.110	16.88	2	TGCAGCGTCGTCGTGGAGGACGGCGACATCGACTTCGTCGTCGCCCAGAGCCCCGTCCTGGAGACACTG	chr2H:18294981–18295049:1	1.1E−30
3255012	2H	12.180	19.06	2	TGCAGCAATATACCACTACTTTGTTTTTTCTTATAACGTGACCTGGCAGTGCTACTAGGACAGGGCCTG	chr2H:18553504–18553572:1	1.1E−30
3255982	2H	13.030	20.57	2	TGCAGAGTTGTGAGTTTGTCCACAAACACCGCGCAAGTAATTATTAGGCACACATATTAGCTAAATAAT	chr2H:21376519–21376587:1	1.1E−30
3257566	2H	17.850	8.40	2	TGCAGCTTCCAGTGGAGGTCCTGGTTGATGAAGCTGAGAGACTCGATGGACGACGCGATGGCGCTGCTC	chr2H:28510043–28510111:-1	2.8E-28
3255272	2H	19.050	6.16	2	TGCAGCATCAATCAGCAATGCACTGAGATAGTAGATATCAGTAGCAATGCAATCAGATGTATGCAAAAT	chr2H:28741801–28741869:-1	1.1E−30
3259351	2H	19.050	5.14	2	TGCAGCTTGGGCCGCCTCGTCTGTCATACTCAAAGCCGAATGCCATCCTCAACGGCGTTGAGGTCATGA	chr2H:28614091–28614159:-1	2.8E−28
3261637	2H	19.050	6.16	2	TGCAGGGCGGTCGTGGAGTTGTACTTCCACACGGCGCCCCGCAACTCCTGCATCGGCTCCCAGTCGTAA	chr2H:28938807–28938875:1	2.8E−28

^1^Kruskal-Wallis K-statistic value, *α* = 0.05; ^2^number of degrees of freedom; ^3^results for BLASTN against the barley genome Hv_IBSC_PGSB_v2 on EnsemblPlants database release 37 (www.plants.ensembl.org, accessed 28.11.2017)

Comparative map analysis revealed eight common DArTseq markers between the AS-DArT and silicoDArT maps and 12 between the silicoDArT and consensus 2H maps (Fig. 1).

### Test of allelism

**Fig. 2:**
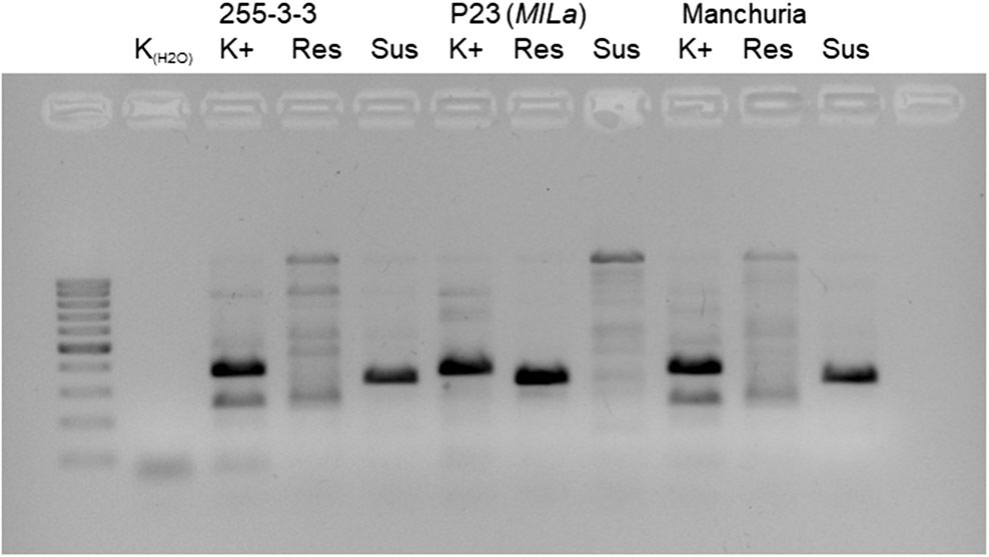
Allele-specific PCR products of Res and Sus variants of marker MWG097 and original MWG097 as a positive control (K+); (K_H2O_)—not template control

An allelic test was performed between the barley 255-3-3 resistance gene and *MlLa*, a powdery mildew resistant gene previously mapped to chromosome 2H (Giese et al. 1993; Hoseinzadeh et al. 2019). A phytopathological test of 315 F_2_ 255-3-3 × P23 (*MlLa*) individuals revealed resistant and susceptible plants. The chi-squared test did not support a 15:1 segregating ratio (Table 2). Analysis of Res (resistant) and Sus (susceptible) AS-PCR variants of MWG097 marker linkages with *MlLa* (Mohler and Jahoor 1996) revealed polymorphism between the parental lines (Fig. 2). Amplification of a Sus variant was obtained for Manchuria and 255-3-3, and amplification of a Res variant was obtained for P23 (*MlLa*).

## Discussion

Investigation of barley landrace genetic resources can broaden the available gene pool. The aim of the present study was to characterise a new source of barley resistance to powdery mildew. The barley line 255-3-3, which was previously described as having broad-spectrum resistance, originates from the Moroccan landrace (Czembor 2002). Czembor (2002) showed that the 255-3-3 line was resistant to all 23 *Bgh* isolates assessed, and exhibited an IT score of 0 according to the Mains and Dietz scale (1930) after inoculation with 17 (81%) isolates. In the present phytopathological tests, 255-3-3 exhibited resistance to all 25 *Bgh* isolates tested (Table 1). Nonetheless, due to the distinctiveness of the 255-3-3 IT spectrum from the spectra of the DV set, representing the majority of known powdery mildew resistance genes introduced into European crops (Dreiseitl 2014), the IT data did not indicate any commonly known resistance genes. This result indicates that 255-3-3 carries unique resistance genes that are not represented in the DV set.

Phenotypic analysis of F_2_ and F_2:3_ progeny of 255-3-3 × Manchuria showed a segregation rate indicating a single dominant resistance gene in the 255-3-3 line.

To reveal the linkage between the resistant gene and genetic location, efforts were focused on homozygous F_2_ individuals. Extreme segregants analysis on the DArTseq platform is an economical and useful method to search for significantly associated markers. DArTseq data processing allowed the construction of two linkage maps. The genetic distance of the *RBgh255* resistance locus to the nearest markers is 5.5 cM on the AS-DArT map and 0.16 cM on the silicoDArT map (Fig. 1). As the genotyping populations comprised 94 and 43 plants, the resulting map densities are satisfactory for determining the *RBgh255* locus. The results provide strong evidence for the presence of *RBgh255* on the distal end of chromosome 2HS, despite divergence between the distances and the order of markers located on the analysis maps, this inconsistency was caused by the small size of the mapping population. In general, a population size ranging from 50 to 250 individuals is sufficient for preliminary genetic mapping (Mohan et al. 1997), although larger mapping populations may close the gap with markers having high linkage and producing more accurate genetic maps (Ferrera et al. 2006).

There are 13 known powdery mildew race-specific resistance genes and one partial resistance *mlo* gene of known position in the barley genome. Among them, only *MlLa* has been mapped to 2H, on the distal end of the long arm (Hoseinzadeh et al. 2019). This powdery mildew resistance gene, which originates from the botanical variety Laevigatum (Giese et al. 1993), was introduced to the Dutch cultivar ‘Vada’ in the 1950s. There is no known major resistance gene for powdery mildew on the short arm of chromosome 2H originating from cultivated barley (Jørgensen and Wolfe 1994; Schönfeld et al. 1996).

An allelic test between *RBgh255* and *MlLa* revealed segregation in the F_2_ generation, with susceptible individuals proving that *MlLa* and *RBgh255* are not allelic variants. The ratio of resistant to susceptible plants within the population deviated from the expected 15:1 for two independent dominant genes, which may be due to some error in the IT rating. *MlLa* is known to confer moderate resistance, corresponding to scores of 2–3 on Mains’ and Dietz’s scale (Marcel et al. 2007), and this level of resistance may cause misclassification of resistant plants as susceptible. Res and Sus variants of the marker MWG097 (Mohler and Jahoor 1996) confirmed different variants at *MlLa* carried by 255-3-3 and P23 (*MlLa*) (Fig. 2). The different IT in interaction with the set of *Bgh* isolates utilised indicated that the resistance gene carried by 255-3-3 is not allelic to *MlLa* (Table 1).

Extensive research on cultivars, landraces and wild barley genotypes revealed resistance loci on 2H. *MlHb*, which is a resistance gene originating from *H. bulbosum*, was mapped to chromosome 2H(2HI) after introgression to *H. vulgare* (Pickering et al. 1995). A strong crossing barrier between bulbous and cultivated barley excludes natural transfer of *MlHb* to *H. vulgare* (Blattner 2018). Nevertheless, Comadran et al. (2009), analysed almost 200 barley accessions from the Mediterranean basin area and indicated coincidence of the *Bgh* resistance gene with the approximate *MlHb* location, which suggested the occurrence of an alternative resistance locus in cultivated barley located on 2H bins: 3, 4 and 5 of the Steptoe × Morex bin map (Kleinhofs and Graner 2001; Druka et al. 2002; Cooper et al. 2004). The distal end of 2H bin 3 was mapped to the MWG878 marker with a physical location of approximately 11 Mb (chr2H:11119104–11119596) according to barley genome Hv_IBSC_PGSB_v2 on the EnsemblPlants database release 43 (www.plants.ensembl.org) (Aken et al. 2017). The *RBgh255* resistance gene is located on 2H bin 1 according to the physical location of DArTseq marker 3255919 with the highest K-statistic (chr2H:1839535:1839604) (Table 4). The proximal end of 2H bin 1 is located on the ABG058 marker with a physical position of 3.2 Mb (chr2H:3239540–3239624). Considering an approximate distance of 10 Mb between *RBgh255* and the candidate genes described by Comadran et al. (2009), there is strong evidence that these loci are different. Spies et al. (2012) mapped candidate genes for resistance to *Bgh* on 2H in barley cv. Steffi. This variety was included in the DV set used in this study and showed distinctive resistance spectrum from 255-3-3 line after inoculation with the *Bgh* set (Table 1). Genetic analysis of Steffi resistance (Spies et al. 2012) indicated quantitative trait segregation and polygenic inheritance that was opposite to the qualitative and monogenic resistance of 255-3-3 line. Previous reports have also revealed *Bgh* resistance loci in wild barley (*H. vulgare* ssp. *spontaneum*) accessions on 2H (Řepková et al. 2009; Tuterová et al. 2010; Ames et al. 2015). Analysis of *H. spontaneum* lines PI282605 (Řepková et al. 2009) and PI466197 (Tuterová et al. 2010) indicated quantitative and semi-dominant loci opposite to those of the qualitative fully dominant *RBgh255*. Furthermore, both described QTL were mapped proximal to *RBgh255,* with the highest associations with Bmac0134 (chr2H:4010391–4010488) (Tuterová et al. 2010) and cMWG682 (chr2H:3326342–3326919) (Řepková et al. 2009) located on 2H bin 2. Since *RBgh255* is located on 2H bin 1, this gene is very unlikely to be the same as the loci described by Řepková et al. (2009) and Tuterová et al. (2010). The powdery mildew QTL described by Ames et al. (2015) was collocated with the *lang1031QPm.S42-2H.a* field resistance QTL (von Korff et al. 2005) and mapped near the HVM36 marker with a physical location of 22 Mb (chr2H:22074463–22074562) on 2H bin 3, which was apart from the *RBgh255* resistance gene. Moreover, QTL corresponding to 2H (Aghnoum et al. 2010) are involved in seedling and adult plant resistance to *Bgh* under field and controlled conditions. Among them, *Rbgq7* carries seedling resistance in controlled conditions but has been mapped on 2H bin 4. These works showed many loci corresponding to 2H as a significant and prospective source for *Bgh* resistance. Differences in phenotypes, inheritances, and location on 2H between *RBgh255* and other described loci indicate that *RBgh255* is probably a distinctive and newly described resistance gene.

Resistance genes have been previously reported in landraces (Comadran et al. 2009; Czembor 2000, 2002; Newton et al. 2000; Spies et al. 2012), and have been successfully introduced into elite germplasms. These genes include *Mlg* originating from the German landrace Weihenstephan, *Mla3* from the Uruguayan landrace Ricardo, *Mla12* from Arabische and the durable resistance recessive gene *mlo*, originating from Ethiopian landraces. The *RBgh255* gene is potentially valuable to breeders for breeding resistance to powdery mildew, and rare broad-spectrum resistance is promising for growers and interesting for scientists. According to the statement “Only when a pathogen isolate with virulence corresponding to that resistance is found can the resistance gene, according to terminology, be classified as race-specific” (Jørgensen and Wolfe 1994), a *Bgh* isolate virulent to 255-3-3 has not been found, in either the previous research by Czembor (2002) or this study. In this work, phytopathological tests were conducted with a *Bgh* set covering 21 common and known resistance genes and 13 *Mla* alleles. However, a limitation of the present study is that it focused only on Polish *Bgh* isolates; conversely, Czembor’s studies, which were conducted more than 15 years ago, utilised isolates from central Europe emerging at that time. New promising resistance genes should correspond to virulence genes in the pathogen population; therefore phytopathological tests were performed using *Bgh* collected from Poland in recent years. The study of markers linked to the identified gene provides a good basis for the development of more useful MAS markers closer to *RBgh255*.

This study revealed and characterised a novel powdery mildew resistance gene in barley line 255-3-3 selected from the Moroccan landrace. In accordance with nomenclature recommendations (Jørgensen 1987), the authors propose *MlMor* as a gene symbol for the resistance described.
